# Aromatic-turmerone induces neural stem cell proliferation *in vitro* and *in vivo*

**DOI:** 10.1186/scrt500

**Published:** 2014-09-26

**Authors:** Joerg Hucklenbroich, Rebecca Klein, Bernd Neumaier, Rudolf Graf, Gereon Rudolf Fink, Michael Schroeter, Maria Adele Rueger

**Affiliations:** Cognitive Neuroscience, Institute of Neuroscience and Medicine (INM-3), Research Centre Juelich, Leo-Brandt-Straße 52425, Jülich, Germany; Department of Neurology, University Hospital of Cologne, Cologne, Germany; Max Planck Institute for Neurological Research, Cologne, Germany

## Abstract

**Introduction:**

Aromatic (ar-) turmerone is a major bioactive compound of the herb *Curcuma longa*. It has been suggested that ar-turmerone inhibits microglia activation, a property that may be useful in treating neurodegenerative disease. Furthermore, the effects of ar-turmerone on neural stem cells (NSCs) remain to be investigated.

**Methods:**

We exposed primary fetal rat NSCs to various concentrations of ar-turmerone. Thereafter, cell proliferation and differentiation potential were assessed. *In vivo*, naïve rats were treated with a single intracerebroventricular (i.c.v.) injection of ar-turmerone. Proliferative activity of endogenous NSCs was assessed *in vivo*, by using noninvasive positron emission tomography (PET) imaging and the tracer [^18^F]-fluoro-L-thymidine ([^18^F]FLT), as well as *ex vivo*.

**Results:**

*In vitro*, ar-turmerone increased dose-dependently the number of cultured NSCs, because of an increase in NSC proliferation (*P* < 0.01). Proliferation data were supported by qPCR-data for Ki-67 mRNA. *In vitro* as well as *in vivo*, ar-turmerone promoted neuronal differentiation of NSCs. *In vivo*, after i.c.v. injection of ar-turmerone, proliferating NSCs were mobilized from the subventricular zone (SVZ) and the hippocampus of adult rats, as demonstrated by both [^18^F]FLT-PET and histology (*P* < 0.05).

**Conclusions:**

Both *in vitro* and *in vivo* data suggest that ar-turmerone induces NSC proliferation. Ar-turmerone thus constitutes a promising candidate to support regeneration in neurologic disease.

## Introduction

Curcumin and ar-turmerone are the major bioactive compounds of the herb *Curcuma longa*. Although many studies have demonstrated curcumin to possess antiinflammatory and neuroprotective properties (reviewed by [1]), to date, the effects of ar-turmerone remain to be elucidated. For example, antitumor properties, exerted via the induction of apoptosis [2] and inhibition of tumor cell invasion [3], have been attributed to ar-turmerone. Park *et al*. [4, 5] recently suggested that ar-turmerone also possesses antiinflammatory properties resulting from the blockade of key signaling pathways in microglia. Because microglia activation is a hallmark of neuroinflammation and is associated with various neurologic disorders, including neurodegenerative diseases [6, 7] and stroke [8, 9], ar-turmerone constitutes a promising therapeutic agent for various neurologic disorders.

The regenerative potential of endogenous neural stem cells (NSCs) plays an important role in neurodegenerative disease and stroke. Endogenous NSCs are mobilized by cerebral ischemia [10] as well as by various neurodegenerative diseases [11, 12], although their intrinsic regenerative response is insufficient to enable functional recovery. The targeted (that is, pharmacologic) activation of endogenous NSCs has been shown to enhance self-repair and recovery of function in the adult brain in both stroke [13, 14] and neurodegeneration [15]. Importantly, NSCs and microglia relevantly interact with each other, thereby affecting their respective functions [16, 17].

Thus, with the perspective of ar-turmerone as a therapeutic option in mind, we investigated the effects of ar-turmerone on NSCs *in vitro* and *in vivo*.

## Material and methods

### Cell culture

NSCs were cultured from fetal rat cortex at embryonic day 14.5, as described previously [18]. Cells were expanded as monolayer cultures in serum-free DMEM/F12 medium (Life Technologies, Darmstadt, Germany) with N2 supplement (Gibco, Karlsruhe, Germany) and fibroblast growth factor (FGF2; 10 ng/ml; Invitrogen, Karlsruhe, Germany) for 5 days and were replated in a 24-well plate at 10,000 cells per cm^2^. FGF2 was included throughout the experiments.

Ar-turmerone (Fluka, Munich, Germany) was added to cultures at replating at concentrations of 0, 1.56, 3.125, 6.25, 12.5, and 25 μg/ml. All experiments were performed in triplicate. After 72 hours, representative pictures were taken by using an inverted fluorescence phase-contrast microscope (Keyence BZ-9000E). Three images were taken per well, and cells were counted by using the software ImageJ with a threshold of 20 px (National Institutes of Health, Bethesda, MD, USA, Version 1.47 k).

To determine the ratio of proliferating cells, 10 μ*M* bromodeoxyuridine (BrdU; Fluka, Munich, Germany) was added to cultures for 6 hours, before cells were fixed with 4% PFA. Again, all experiments were performed in triplicate. Cells were stained with mAb against BrdU to identify proliferating cells (clone BU-33, dilution 1:100; Sigma-Aldrich, Munich, Germany). For antigen-retrieval before staining, sections were incubated in 2 N HCl for 30 minutes. For visualization, FITC-labeled anti-mouse IgG was used (Invitrogen); all cells were additionally counterstained with Hoechst 33342 (Life Technologies). To calculate the ratio of proliferating cells, BrdU-positive cells were divided by the total cell number in each sample, and mean values were established among equally treated cells.

To establish its effect on cell survival, ar-turmerone was added to NSC cultures for 24 hours. To discriminate between live and dead cells, the live/dead cell-mediated cytotoxicity kit (Life Technologies, cat. no. L7010) was used according to the manufacturer’s instructions. Both viable and dead NSCs were counted in *n* = 6 samples per condition, and a ratio of surviving cells was calculated for each field of view; mean values were calculated for each concentration tested.

To assess the differentiation potential of NSCs treated with ar-turmerone, mitogen was withdrawn during the expansion phase, followed by a differentiation phase of 10 days, in the absence (control) or presence of 6.25 μg/ml ar-turmerone. Immunocytochemistry with markers for young neurons (TuJ1), astrocytes (GFAP), and oligodendrocytes (CNPase) was used to verify all three differentiated fates of NSCs, whereas SOX2 marked undifferentiated NSCs.

### Real-time quantitative PCR (RT-qPCR)

RNA from cells was isolated by using the RNeasy Mini Kit (Qiagen, Hilden, Germany). Total RNA concentration and purity were evaluated photometrically. Total RNA was converted to c-DNA by reverse transcription with the Quantitect reverse transcription kit (Qiagen). The primer used for Ki67 was obtained from Biolegio (Nijmegen, The Netherlands). The sequences of the primers were as follows: (a) forward: TCTTGGCACTCACAGTCCAG, and (b) reverse: GCTGGAAGCAAGTGAAGTCC. The q-PCR reaction was carried out by using 10 ng total RNA in a 20-μl reaction (Quantitect Reagents, Qiagen) according to the manufacturer’s instructions. The samples were amplified and quantified on a Rotorgene 2000 (Corbett, Sydney, Australia) by using the following thermal cycler conditions: activation: 95°C 10 minutes; cycling: 50 cycles, step 1: 92°C, 15 seconds, step 2: 52°C, 15 seconds, and step 3: 72°C, 40 seconds. PCR product integrity was evaluated by melting-point analysis and agarose gel electrophoresis. Each sample and gene was normalized to RPL13a as reference gene [19]. Ki67 mRNA levels were normalized to endogenous RPL13a expression (ΔCT); normalized values were then expressed as 2^-ΔCt^. Mean values were calculated for treated and untreated cells.

### Animals and surgery

All animal procedures were in accordance with the German Laws for Animal Protection and were approved by the local animal care committee (Buero der Tierschutzbeauftragten, MPIfNF, Cologne, Germany), as well as local governmental authorities (LANUV NRW 84–02.04.2012.A116). Spontaneously breathing male Wistar rats weighing 290 to 330 g were anesthetized with 5% isoflurane and maintained with 2.5% isoflurane in 65%:35% nitrous oxide/oxygen. Throughout surgical procedures, the body temperature was maintained at 37.0°C with a thermostatically controlled heating pad.

#### Intracerebroventricular injections

One group of animals (*n* = 3) underwent a single intracerebroventricular (i.c.v.) injection of 3 mg ar-turmerone at a concentration of 1 mg/μl. For control, *n* = 6 rats were vehicle-injected with the identical volume of normal saline. Under anesthesia with 1.5% isoflurane, each rat’s skull was fixated in a stereotaxic frame in plane orientation. After incision of the skin, the bregma was exposed, and a burr hole was drilled over the right lateral ventricle by using the following stereotaxic coordinates: bregma, AP −0.9 mm; ML, −1.4 mm; and VD, +3.8 mm. Ar-turmerone dissolved in normal saline, or respectively, pure saline as control, was injected at 1 μl/min. After injection, the needle was left in place for another 5 minutes to allow a distribution of the solution within the ventricles. The needle was thereafter withdrawn slowly, and the skin sutured with nonabsorbing silk.

After each procedure, all animals were allowed to recover from anesthesia and were put back into their home cages, where they were given access to food and water *ad libitum*.

#### BrdU injections

In all animals, the tracer bromodeoxyuridine (BrdU) was injected intraperitoneally for 5 days, starting on the day of i.c.v. injection, at a concentration of 50 mg/kg per injection, as described previously [18]. This regimen resulted in a cumulative dose of 250 mg/kg BrdU per animal.

### Positron emission tomography (PET)

[^18^F]-fluoro-L-thymidine ([^18^F]FLT) was synthesized as described previously [20]. Seven days after i.c.v. injection of ar-turmerone or placebo, respectively, PET imaging was performed on a microPET Focus 220 scanner (Concorde Microsystems, Inc., Knoxville, TN, USA; 63 image planes; 1.5-mm full width at the half maximum). Animals were anesthetized with 5% isoflurane, maintained with 2% isoflurane in a 65%:35% nitrous oxide/oxygen atmosphere, and placed in the scanner. Temperature was monitored by using a rectal probe and maintained at 37°C ± 0.5°C by a thermostatically controlled water-flow system (Medres, Cologne, Germany). After a 10-minute transmission scan for attenuation correction, rats received an intravenous bolus injection of [^18^F]FLT (1.0 to 2.2 mCi/rat), and emission data were acquired for 60 minutes. PET data were reconstructed in two time frames of 1,800 seconds. The last frame (that is, minutes 31 to 60 after tracer injection) was used for image analysis.

### Image analysis

PET images were co-registered to anatomic data of a 3D rat-brain atlas constructed from the brain slices presented by Swanson [21]. Based on the 3D anatomic data, ellipsoid volumes of interest (VOIs) measuring 4 mm^3^ were placed to cover the subventricular zone (SVZ) as well as the dentate gyrus region of the hippocampus. A standard uptake value (SUV) was calculated for each VOI, dividing maximal VOI activity by the decay-corrected injected radioactive dose per body weight. SUVs were individually determined and then averaged between animals within each group.

### Immunohistochemistry

After PET imaging, or 7 days after ar-turmerone treatment, rats were deeply anesthetized and decapitated. The brains were rapidly removed, frozen in isopentane, and stored at −80°C before further histologic and immunohistochemical processing. Ten-μm-thick adjacent serial coronal brain sections were cut at 500-μm intervals and stained with anti-BrdU to identify proliferating cells (mAb clone BU-33, dilution 1:200; Sigma-Aldrich), or with anti-doublecortin (DCX) to identify neuroblasts (rabbit polyclonal, dilution 1:1,000, Sigma-Aldrich). For antigen-retrieval before BrdU staining, sections were microwave-heated in 0.01 *M* citrate buffer, pH 6.0, for 5 minutes, followed by 2 N HCl at 37°C for 30 minutes. For visualization, the ABC Elite kit (Vector Laboratories with diaminobenzidine (Sigma-Aldrich) as the final reaction product was used.

To quantify the width of the SVZ and of the dentate gyrus of the hippocampus, it was measured on three consecutive BrdU-stained slices per animal, and an average was calculated per animal. To quantify the number of neuroblasts in the SVZ, their number was counted on three consecutive DCX-stained slices within a standardized field-of-view for each animal. For both schemes of quantification, mean values were calculated for each group of animals.

### Statistical analysis

Descriptive statistics were performed with Microsoft Excel 2003 (Microsoft Corp., Redmond, WA, USA). One-way ANOVA tests (followed by Holm-Sidak *post hoc* test) were performed with SigmaPlot 11.0 for Windows (Systat Software Inc., San Jose, CA, USA). Statistical significance was set at *P* < 0.05.

## Results

### Effects on NSC proliferation *in vitro*

**Figure 1:**
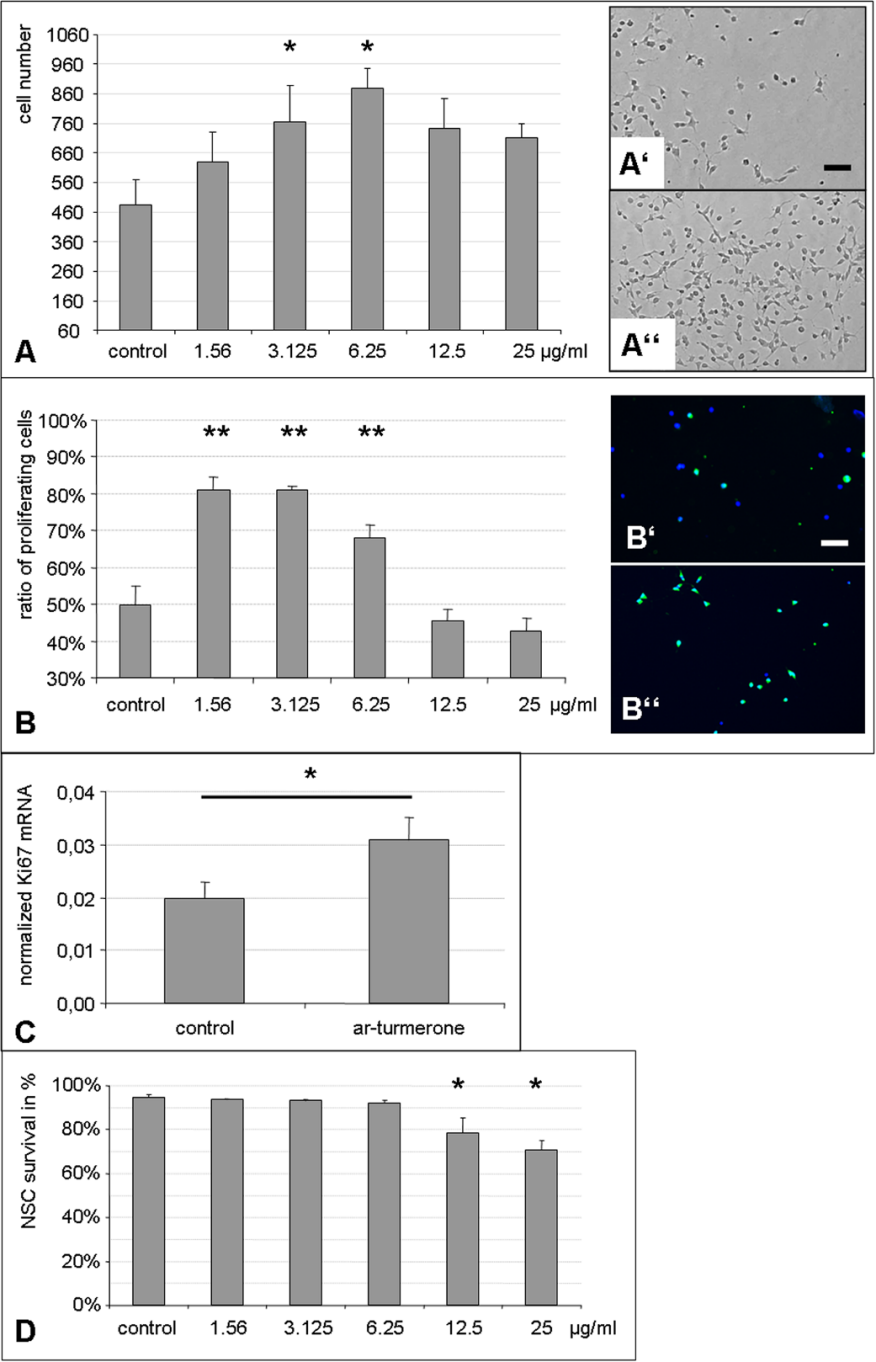
**Ar-turmerone increases NSC proliferation** ***in vitro.*** **(A)** Ar-turmerone significantly increased the numbers of fetal rat NSCs in primary monolayer culture (mean ± SEM; *P < 0.05, compared with control), dependent on its concentration; representative phase-contrast images are depicted of NSC-treated without (Aʹ) or with (Aʹʹ) 6.25 μg/ml ar-turmerone (bar represents 200 μm). **(B)** Ar-turmerone significantly increased the number of proliferating NSCs, as assessed by BrdU-incorporation (mean ± SEM; **P < 0.01, compared with control), dependent on its concentration; representative images are depicted of NSCs treated without (Bʹ) or with (Bʹʹ) 3.125 μg/ml ar-turmerone, stained for BrdU-incorporation (bar represents 200 μm). **(C)** Treating NSCs with 6.25 μg/ml ar-turmerone led to a significant increase in Ki67 mRNA; mRNA levels were normalized to endogenous RPL13a expression and calculated with the 2^-ΔCt^ method; data are depicted as mean ± SEM; *P < 0.05. **(D)** In high concentrations, ar-turmerone significantly decreased ratio of surviving NSCs within 24 hours of treatment, wheres concentrations between 1.56 and 6.25 μg/ml had no effect (mean ± SEM; *P < 0.05, compared with control).

To assess the effects of ar-turmerone on NSC in primary culture, rat fetal NSC were grown in the presence of various concentrations of ar-turmerone for 72 hours. Cell numbers significantly increased when NSCs were treated with 3.125 to 25 μg/ml ar-turmerone (*P* < 0.05), with a maximum increase of NSC numbers by ~80% at 6.25 μg/ml (Figure 1A; *P* < 0.01).

With the BrdU-incorporation assay, we next investigated whether this increase in NSC number was caused by an increase in NSC proliferation. Indeed, treatment with certain concentrations of ar-turmerone significantly increased the percentage of proliferating NSCs from ~50% to ~80% (Figure 1B; *P* < 0.01). This result was verified on the mRNA level by using qPCR for the proliferation marker Ki67. In line with the BrdU data, treatment with 6.25 μg/ml ar-turmerone led to a significant increase in Ki67 mRNA (Figure 1C; *P* < 0.05).

To assess whether ar-turmerone affected NSC survival, viable and dead cells were determined after 24 hours, and the proportion of surviving cells was quantified for each concentration of ar-turmerone. Concentrations between 1.56 and 6.25 μg/ml that had yielded the maximum effect on NSC proliferation did not affect cell survival. Higher concentrations of 12.5 and 25 μg/ml led to a significant decrease in the number of viable NSCs (Figure 1D; *P* < 0.05).

### Differentiation potential of NSCs

**Figure 2:**
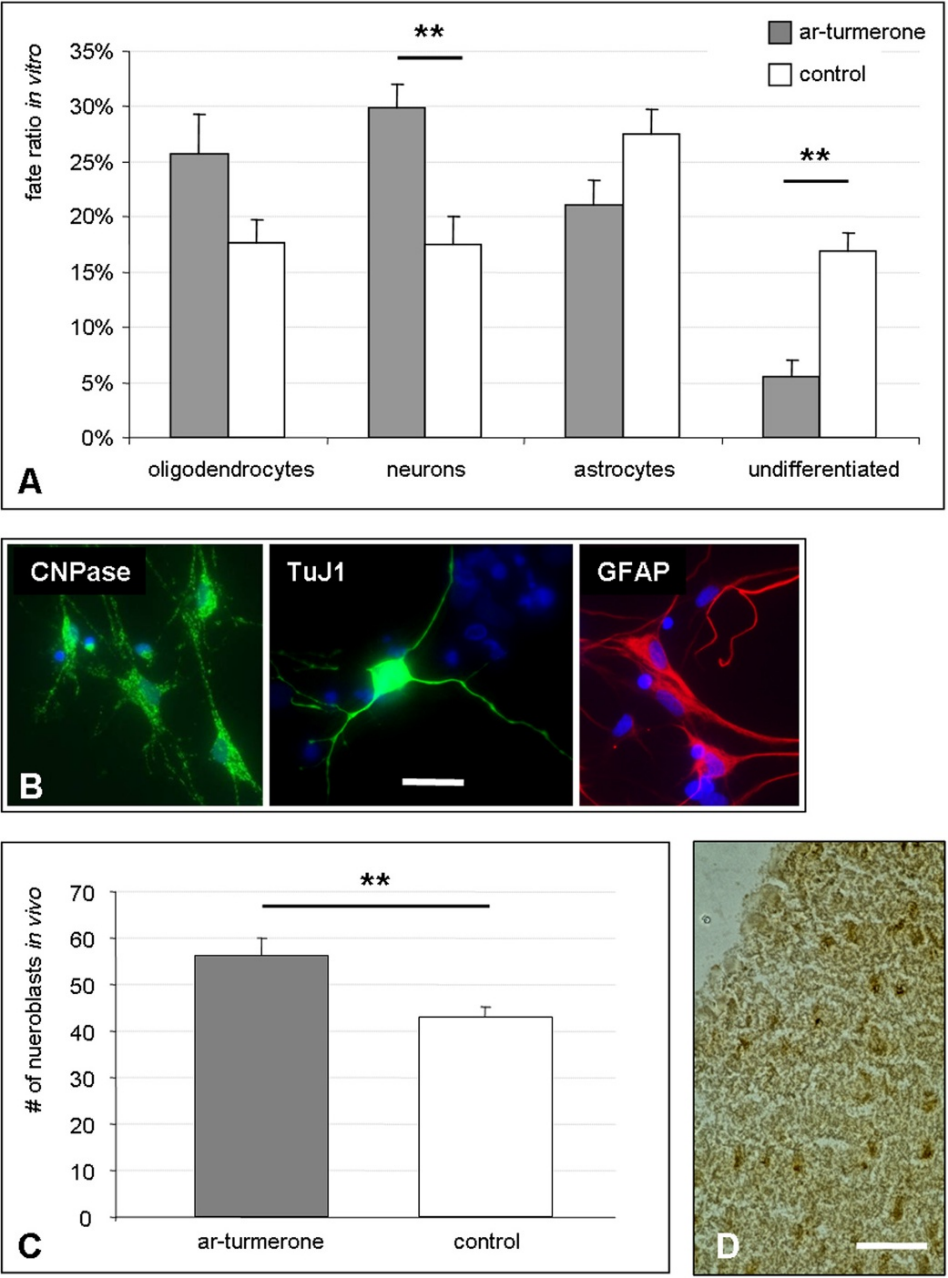
**Ar-turmerone induces neurogenesis** ***in vitro*** **and** ***in vivo.*** **(A)** NSCs were allowed to differentiate in the absence (control) or presence of 6.25 μg/ml ar-turmerone. Immunocytochemistry 10 days after growth-factor discontinuation revealed fewer undifferentiated (SOX2+) NSCs in the turmerone-treated group, but more young neurons. The generation of astrocytes and oligodendrocytes was not affected by ar-turmerone (mean ± SEM; **P < 0.01, compared with control). **(B)** Representative images of differentiated cells include CNPase-positive oligodendrocytes (left), TuJ1-positive young neurons (middle), and GFAP-positive astrocytes (right); bar represents 50 μm. **(C)** After i.c.v. injection of 3 mg (1 mg/μl) ar-turmerone, significantly more DCX-positive neuroblasts were observed in the SVZ compared with placebo-injected control animals (mean ± SEM; **P < 0.01). **(D)** Representative staining of DCX-positive neuroblasts in the SVZ (bar represents 50 μm).

To assess the effect of ar-turmerone on the differentiation potential of NSCs *in vitro*, cells in the expansion phase were treated with or without 6.25 μg/ml ar-turmerone and allowed to differentiate for 10 days by withdrawal of FGF2. Compared with that in untreated control cells, the differentiation process was significantly accelerated in ar-turmerone-treated NSCs, with fewer undifferentiated (SOX2-positive) cells 10 days after FGF2-withdrawal (Figure 2A; *P* < 0.01). Moreover, ar-turmerone-treated NSCs preferentially differentiated into young neurons, as assessed by TuJ1 staining, compared with untreated control cells (Figure 2A, B; *P* < 0.01). The generation of GFAP-positive astrocytes and CNPase-positive oligodendrocytes was unaffected by ar-turmerone (Figure 2A, B).

To investigate the effects of ar-turmerone on neurogenesis *in vivo*, adult rats were injected with 3 mg ar-turmerone into the lateral ventricle of the brain (intracerebroventricular, i.c.v.). One week after treatment, the number of DCX-positive neuroblasts in the subventricular zone (SVZ) was significantly increased compared with placebo-injected control animals (Figure 2C, D).

### Proliferation of endogenous NSCs *in vivo*

**Figure 3:**
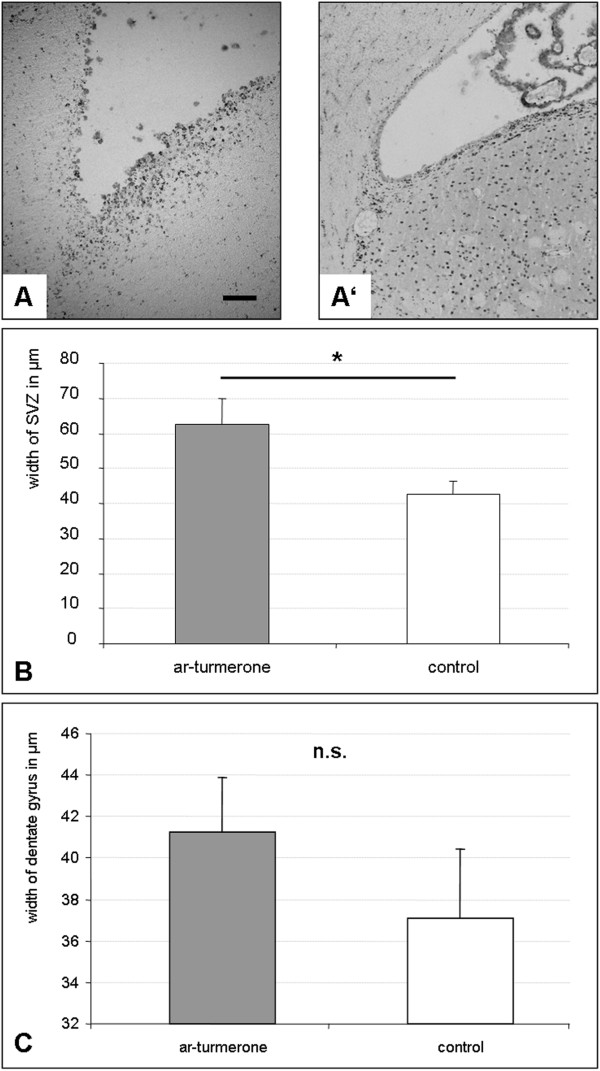
**Proliferation of endogenous NSC is induced by ar-turmerone** ***in vivo.*** **(A)** Staining for proliferating NSCs with anti-BrdU demonstrates that the subventricular zone (SVZ) of rats treated with 3 mg (1 mg/μl) ar-turmerone i.c.v. (left) was wider than that of placebo-treated control animals (Aʹ, right); bar represents 100 μm. **(B)** Differences in the width of the SVZ were statistically significant (mean ± SEM; *P < 0.05, compared with control). **(C)** BrdU staining of the hippocampus did not reveal a statistically significant increase in the width of the dentate gyrus, although a trend was noted favoring ar-turmerone (mean ± SEM).

The effect of ar-turmerone on endogenous NSCs *in vivo* was assessed by injecting adult rats with ar-turmerone i.c.v. For the following 5 days, rats received daily systemic injections of BrdU to label proliferating cells *in vivo*. Immunohistochemistry 1 week after ar-turmerone treatment revealed the SVZ of treated rats to be wider than that of placebo-injected control animals, as measured by BrdU staining (Figure 3A). Differences in the size of the SVZ, as assessed by BrdU-staining, were statistically significant (Figure 3B; *P* < 0.05). BrdU-staining of the hippocampus did not reveal a statistically significant increase in the width of the dentate gyrus, although a trend was noted toward a wider dentate gyrus after treatment with ar-turmerone (Figure 3C).

### Mobilization of endogenous NSCs from the neurogenic niches

A noninvasive PET-imaging assay was used to visualize and quantify the mobilization of endogenous NSCs from the neurogenic niches of ar-turmerone-treated animals *in vivo*. One week after i.c.v. injection of ar-turmerone, the radiotracer [^18^F]FLT was injected systemically to label proliferating endogenous NSCs *in vivo*, and then PET data were acquired and co-registered to a 3D rat brain atlas.

**Figure 4:**
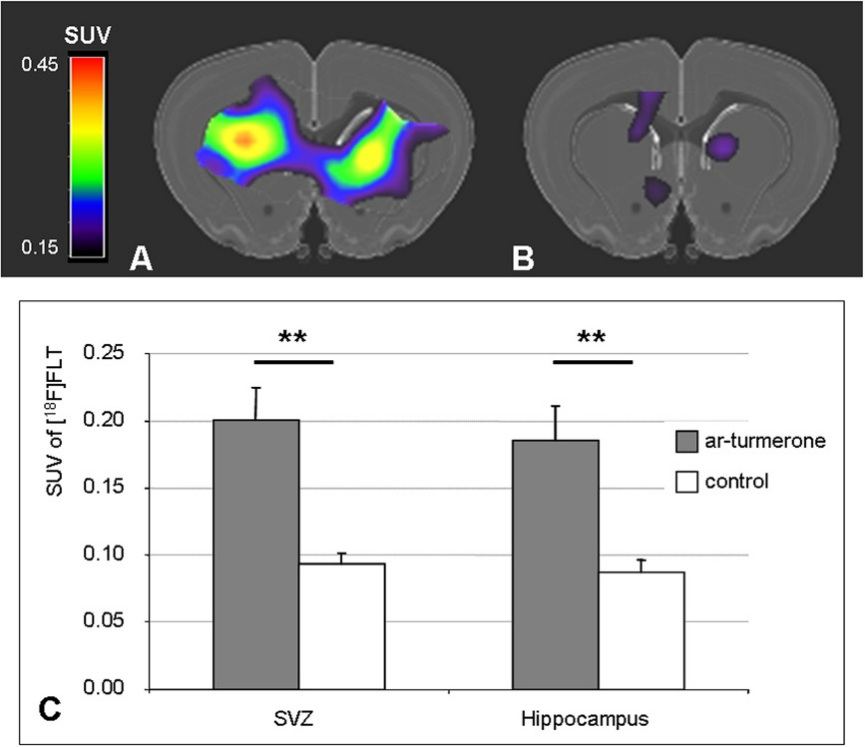
**Endogenous NSCs in the neurogenic niches of the rat brain are mobilized by ar-turmerone** ***in vivo.*** **(A)** [^18^F]FLT-PET of a rat brain 1 week after intracerebroventricular injection of ar-turmerone shows enhanced accumulation of [^18^F]FLT in the subventricular zone compared with **(B)** Saline-injected control brain, indicating an increase of proliferating endogenous NSCs caused by ar-turmerone. **(C)** Ar-turmerone-treated rats showed significantly more [^18^F]FLT accumulation in the SVZ and the hippocampus than did control animals (mean ± SEM; **P < 0.01).

The brains of ar-turmerone-treated rats showed marked accumulation of [^18^F]FLT in the SVZ ipsi- and contralateral to the i.c.v. injection (Figure 4A), compared with saline-injected control animals (Figure 4B). Moreover, ar-turmerone-treated rats showed significantly more [^18^F]FLT-accumulation in both the SVZ and the hippocampus than the control animals (*P* < 0.01), thus indicating a mobilization of proliferating NSCs from both neurogenic niches (Figure 4C).

## Discussion

The data suggest that ar-turmerone increases the proliferative activity of NSCs. Recently, both positive and negative effects on proliferation have been attributed to ar-turmerone, dependent on the cell type studied [22]. Although ar-turmerone inhibited the proliferation of various cancer cell lines, it enhanced proliferation of peripheral blood mononuclear cells [22]. With the prospect of evaluating ar-turmerone as a drug candidate for neurodegenerative disorders or stroke, one must keep in mind that enhancing the proliferation of NSCs, especially by genetic manipulations, bears a certain oncogenic risk [23]. However, pharmacologic expansion of the stem cell niche without genetic manipulations seems to be less associated with an increased cancer risk [13, 14].

Another issue to be considered before promoting the use of ar-turmerone in clinical studies is that we here applied ar-turmerone *in vivo* via i.c.v. injection, a route that is obviously not applicable in clinical studies. However, another recent study found good bioavailability of ar-turmerone after both intravenous or intraperitoneal injection in the mouse [24].

*In vivo*, ar-turmerone expanded the width of the SVZ by ~45%. An expansion of this NSC niche has also been demonstrated for other pharmacologic agents such as growth factors [13, 14]. In a similar experimental setting, we previously observed that FGF2 expanded the SVZ by ~350%, whereas a combination of the Notch ligand Delta-like 4 and insulin led to an increase in the width of the SVZ of ~66% [18]. Our data therefore suggest that the effect of ar-turmerone on the NSC niche *in vivo* is somewhat smaller than that of ”classic” NSC-activation pathways. Nevertheless, the pleiotropic effects of ar-turmerone render it a promising drug for further studies.

Ar-turmerone was recently described to inhibit the LPS- or Aß-induced activation of microglia through inhibition of NF-κB, JNK-, and p38-MAPK pathways [4, 5]. Microglia activation as the hallmark of an innate inflammatory response of the central nervous system (CNS) has been found in many neurologic disorders that are considered to be primarily nonimmunogenic, such as stroke [25, 26], traumatic brain injury (TBI [27], Parkinson disease [6], or Alzheimer disease [7]. NSC and immune cells interact extensively [16, 17, 28–30]. Therefore, therapeutically regulating one entity’s fate is likely to influence the other.

Yet, our knowledge about the interaction of NSCs and inflammatory responses in the CNS with regard to regeneration and functional recovery to date remains scarce. On attraction by proinflammatory cytokines, endogenous NSCs considerably affect this regenerative response [31, 32], for example, through inducing remyelinization [33] and neuroprotection [15]. As ar-turmerone both limits microglia activation and induces NSC proliferation, it constitutes a promising future drug candidate to support regeneration in neurologic disorders.

In the presence of mitogen in cell culture, as well as under physiological conditions *in vivo*, we found ar-turmerone to promote neurogenesis. However, after FGF2 discontinuation *in vitro*, treatment with ar-turmerone led to an accelerated decrease of undifferentiated NSC, indicating an early exit from the cell cycle. This effect suggests that ar-turmerone may act as a weak antagonist on the FGF-receptor only in the absence of the ligand.

Further studies are needed to clarify such a putative relationship. In support of this notion, a recent report suggests that ar-turmerone acts as an antagonist on the related epidermal growth factor (EGF) receptor [34].

Noninvasive *in vivo* imaging is a crucial tool for translation from bench to bedside (that is, from experimental animal to human studies). We used PET imaging and the radiotracer [^18^F]FLT that enables imaging and measuring of proliferation, thereby allowing noninvasive detection and quantification of endogenous NSC mobilization in the adult rat brain *in vivo* [18]. This imaging assay is capable of monitoring the effects of drugs aimed at expanding the NSC niche [35]. By using [^18^F]FLT-PET, we here found ar-turmerone to mobilize NSCs from both neurogenic niches, the SVZ and the dentate gyrus of the hippocampus, *in vivo*. Thus, this study provides further evidence for NSC activation by ar-turmerone, spanning from cell-culture findings to *in vivo* imaging.

## Conclusions

In this study, we investigated the effects of ar-turmerone on NSCs *in vitro* and *in vivo*. Ar-turmerone increased the number of NSCs both in cell culture and in the adult rat brain *in vivo.* This increase resulted from enhanced NSC proliferation and led to promoted neurogenesis during differentiation. *In vivo*, ar-turmerone mobilized endogenous NSCs from both neurogenic niches, the SVZ and the hippocampus. We propose that ar-turmerone constitutes a promising future drug candidate to support regeneration in neurologic disorders.

## Abbreviations

[^18^F]FLT: [^18^F]-fluoro-L-thymidine
ar-turmerone: aromatic turmerone
BrdU: bromodeoxyuridine
CNPase: 2´,3´-cyclic nucleotide 3´-phosphodiesterase
DCX: doublecortin
DMEM: Dulbecco Modified Eagle Medium
FGF2: fibroblast growth factor 2
GFAP: glial fibrillary acidic protein
HCl: hydrogen chloride
i.c.v.: intracerebroventricular
NSC: neural stem cell
PCR: polymerase chain reaction
PET: positron-emission tomography
RNA: ribonucleic acid
SUV: standard uptake value
SVZ: subventricular zone
TuJ1: neuron-specific class III beta-tubulin
VOI: volume of interest.
